# Diet, a new target to prevent depression?

**DOI:** 10.1186/1741-7015-11-3

**Published:** 2013-01-03

**Authors:** Almudena Sanchez-Villegas, Miguel A Martínez-González

**Affiliations:** 1 Deparment of Clinical Sciences, University of Las Palmas de Gran Canaria, PO Box 550, CP 35080, Las Palmas de Gran Canaria, Spain; 2 Department of Preventive Medicine and Public Health, University of Navarra, C/Irunlarrea 1, CP 31008, Pamplona, Spain

**Keywords:** Cohort study, dietary patterns, Mediterranean diet, omega-3, trans fatty acids, nutrigenetics, primary prevention trial

## Abstract

**Background:**

Research on the role of diet in the prevention of depression is scarce. Some evidence suggests that depression shares common mechanisms with cardiovascular disease.

**Discussion:**

Before considering the role of diet in the prevention of depression, several points need to be considered. First, in general, evidence has been found for the effects of isolated nutrients or foods, and not for dietary patterns. Second, most previous studies have a cross-sectional design. Third, information is generally collected though questionnaires, increasing the risk of misclassification bias. Fourth, adequate control of confounding factors in observational studies is mandatory.

**Summary:**

Only a few cohort studies have analyzed the relationship between overall dietary patterns, such as the Mediterranean diet, and primary prevention of depression. They have found similar results to those obtained for the role of this dietary pattern in cardiovascular disease. To confirm the findings obtained in these initial cohort studies, we need further observational longitudinal studies with improved methodology, as well as large randomized primary prevention trials, with interventions based on changes in the overall food pattern, that include participants at high risk of mental disorders.

## Background

The prevention of mental disorders is a priority due to their huge health, social and economic burden. Among them, unipolar major depression is the worldwide leading cause of years of healthy life lost as a result of disability [1] and it is projected to also be the leading cause of disability-adjusted life years lost in 2030 [2]. Surprisingly, relatively little etiological longitudinal research has been conducted to assess which are the dietary or lifestyle determinants of depression. In this context, dietary factors are likely to play a major role. Whereas the role of diet in the prevention of other noncommunicable diseases, such as cardiovascular disease (CVD), has been widely investigated for the last 50 years, the relationship between diet and depression is so far a novel and interesting field that has only emerged in the last five to ten years.

### Depression, cardiovascular disease, metabolic syndrome and obesity

There is an accrual of studies suggesting that depression seems to share common mechanisms with the metabolic syndrome (MetS), obesity and CVD. In fact, several major cardiovascular risk factors (including obesity and MetS) are more prevalent among patients who are depressed [3]. Metabolic and inflammatory processes, such as reduced insulin sensitivity, elevations in plasma homocysteine levels and, more importantly, increased production of proinflammatory cytokines and endothelial dysfunction, may be responsible for the link between depression and cardiometabolic disorders [4-6].

Proinflammatory cytokines production interferes with neurotransmitters metabolism and decreases the availability of some precursors such as tryptophan [7]. Moreover, low-grade inflammatory status and endothelial dysfunction inhibit the expression of brain-derived neurotrophic factor (BDNF) because it is the endothelial cells that synthesize and secrete BDNF. An emerging concept in neuroscience is that perturbations in the health of the cerebral endothelium (including some loss of the neuroprotection afforded by BDNF) may mediate progressive neuronal dysfunction [8]. Indeed, results of several meta-analyses have established that BDNF levels are reduced in patients with depression, and that antidepressant medication seems to up-regulate their levels [9,10].

## Discussion

### The role of diet in depression: nutrients or foods versus dietary patterns

To date, most of the evidence relating diet to depression is similar to that demonstrating the role that diet plays on MetS or CVD. This is reasonable, because both diseases seem to share several common physiopathological mechanisms. This analogy is supported by the beneficial effects reported for lipids with anti-inflammatory properties, such as omega-3 fatty acids or olive oil [11,12]. Conversely, the intake of trans fatty acids or the consumption of foods rich in this kind of fat, like fast food or commercial bakery products, have recently been reported as contributors to higher depression risk [12-14]. The mediators of adverse effects of trans fatty acids on CVD include increases in plasma concentrations of low density lipoprotein -cholesterol, reductions in high density lipoprotein -cholesterol, proinflammatory changes, and endothelial dysfunction. Because depression is also associated with a low-grade inflammatory status, endothelial dysfunction and worse lipid profiles, the adverse biological modifications caused by trans fatty acids could also be responsible for detrimental effects on depression.

However, it is more important to study the overall dietary pattern than isolated nutrients. In this context, it is reasonable to think that dietary patterns that foster cardiometabolic health could also be inversely related to depressive disorders. Similarly, those dietary patterns directly involved in cardiometabolic risk could also exert a detrimental effect on depression. A few epidemiological studies have inversely related healthy dietary patterns, including the Mediterranean Diet, or directly related the Western dietary pattern to the risk of developing depression [15-19]. Significant differences in plasma BDNF levels have been observed for patients with depression who were assigned to the Mediterranean diet compared with those assigned to a control diet [20]. Nevertheless, these evidences are sparse and not definitive, because some of these studies were not well-protected against diverse sources of bias.

### Epidemiological evidences: strengths and limitations

Some of the reported associations between diet and depression have been found in studies with large sample sizes. These large studies generally use questionnaires to collect information on the outcome (depression) and/or the exposure (diet). Food frequency questionnaires have been customarily used, but they are known to have some potential for misclassification bias. The use of questionnaires adequately validated in the country where the study was performed is encouraged to minimize misclassification biases. Depression assessment is usually based on depressive symptoms scales. Very often this information is self-reported. Additionally, the choice of a cut-off point to define depression is generally arbitrary. This cut-off point usually depends on the sample characteristics and limits the ability to conduct comparisons between studies carried out in different populations. Thus, the use of medical diagnoses of depression ascertained via clinical assessments or the use of a validated self-reported medical diagnosis of depression could be the most appropriate approach to reduce misclassification problems in large epidemiological studies.

Most of the evidence suggesting a link between nutrition and depression comes from studies with a cross-sectional design. This design usually precludes the possibility to infer a truly causal relationship. In these studies exposure is ascertained simultaneously with disease and, therefore, results could be alternatively interpreted as a consequence of reverse causation bias, that is, depression may lead to poorer dietary habits. Beyond cross-sectional studies, only a few longitudinal studies have prospectively analyzed the role of diet on depression risk. One of these epidemiological studies is the Seguimiento Universidad de Navarra (SUN) Project, a dynamic prospective cohort of university graduates, with a median follow-up of 6 years and the ability to include more than 10,000 participants in longitudinal assessments. Several diet components have been prospectively associated with depression risk in this cohort. Whereas trans fatty acids or fast food and commercial bakery products were associated with higher depression risk [12,13], omega-3 fatty acids and olive oil intake showed inverse associations [11,12]. Moreover, the SUN Project reported in 2009 that better adherence to the traditional Mediterranean dietary pattern was associated with substantially reduced depression risk [15]. Almost immediately after, investigators from the Whitehall II longitudinal study (another prospective cohort in the UK) reported a detrimental role for a Western dietary pattern [16]. Recently, the divergent roles of healthy or Western dietary patterns on depression risk have been confirmed in a longitudinal analysis of Australian adolescents [17]. Other studies conducted on adults in Australia (Geelong Osteoporosis Study) and Norway (Hordaland Health Study) reported similar associations between dietary patterns and depression risk [18,19], but they were based on cross-sectional assessments. Thus, these initial findings need to be confirmed (ideally in these same cohorts) in future prospective assessments.

Finally, the potential effect of dietary patterns on depression could be in part explained by the co-occurrence of other lifestyle-related factors such as physical activity, alcohol intake, smoking or the use of illicit drugs; by sociodemographic factors such as social networks, marital status or socioeconomic level; or by medical conditions such as the presence of CVD. Thus, one of the most important aspects in observational epidemiology is to obtain an adequate control of these possible confounders. Most of these confounders are usually collected in well-designed epidemiological studies and controlled using multivariable models. Restriction is an even better procedure (at least as a sensitivity analysis) that is used on occasion. This procedure consists of excluding all participants with the presence of the confounding condition (that is, cases of prevalent CVD) from the database before assessing the role of diet on incident depression. Nevertheless, when the lack of or inadequate control for some of these potential confounders and the presence of residual confounding exist, the interpretation of the findings obtained from observational studies demands caution.

### Prevention or treatment

Whereas the above-mentioned studies have analyzed the role of diet in the primary prevention of depression, clinical trials have been generally designed to assess the impact of nutritional interventions on the clinical course of depression. However, most available trials are based on small samples and have been carried out in a controlled clinical setting with a short follow-up period. Moreover, with the exception of a recent clinical trial [21], none of these trials has analyzed the effect of an overall dietary pattern. Instead they have assessed isolated nutrients, mainly omega-3 fatty acids or B vitamins [22,23].

### Interaction between diet and genetic factors

To date, there are no studies that ascertain the possible interaction between diet and genetic factors on depression risk. Nevertheless, effect modification of genetic factors by diet on several diseases potentially related to depression, such as obesity or CVD, has been increasingly reported [24,25]. This new line of nutrigenetics research based on the hypothesis that visceral obesity or MetS share some etiological mechanisms, including diet and genes, with unipolar depressive disorder should be developed in the near future. This would help to better understand the role of diet on the risk and prognosis of major depression.

### Future research directions

Large randomized primary prevention trials with interventions based on changes in the overall food pattern and including participants at high risk of mental disorders could provide the most definitive answer to confirm or refute experimentally the findings reported by observational studies. Though ideal, these trials might not seem feasible. However, similar trials have been successfully conducted in cardiovascular fields, as it has been the case for the Dietary Approaches to Stop Hypertension (DASH) diet [26] or the Prevención con Dieta Mediterránea (PREDIMED) trial [27]. Why cannot similar trials be designed for the primary prevention of depression?

## Summary

Although a few prospective cohort studies have analyzed the role of dietary patterns on depression risk, their contributions are still scarce. Further observational studies with improved methodology (including repeated measurements of diet, better validation of measuring instruments, longer follow-up periods, larger sample sizes and adequate control of confounders) as well as large randomized primary prevention trials with interventions based on changes in the overall food pattern and including participants at high risk of mental disorders are necessary to confirm the findings obtained in these initial studies.

## Abbreviations

BDNF: brain derived neurotrophic factor; CVD: cardiovascular disease; DASH: Dietary Approaches to Stop Hypertension; MetS: metabolic syndrome; PREDIMED: Prevención con Dieta Mediterránea; SUN: Seguimiento Universidad de Navarra.

## Competing interests

The authors declare that they have no competing interests.

## Authors' contributions

ASV wrote the manuscript drafts. MAMG read and critically revised manuscript drafts. Both authors read and approved the final manuscript.

## Authors' information

ASV is an Associate Professor of Preventive Medicine and Public Health at the University of Las Palmas of Gran Canaria. She has been funded with several grants from the Instituto De Salud Carlos III, Official Agency of the Spanish Government for biomedical research to analyze the role of diet in depression in the SUN Project. MAMG is a Professor of Preventive Medicine and Public Health at the University of Navarra and the director of the SUN Project.

## Pre-publication history

The pre-publication history for this paper can be accessed here:

http://www.biomedcentral.com/1741-7015/11/3/prepub
